# Foliar and Root Dynamic Analyses Reveal a Coordinated Defense Regulatory Network Against *Aeolesthes induta* Infestation in Tea Plants

**DOI:** 10.3390/insects17090911

**Published:** 2026-09-01

**Authors:** Chengcong Lu, Jialin Zhang, Ke Chen, Xuanyi Zhang, Xi Du, Fajie Feng, Pumo Cai, Yongcong Hong

**Affiliations:** 1 College of Tea and Food Science, Wuyi University, Wuyishan 354300, China; lccfafu@126.com (C.L.); 13656031826@163.com (K.C.); 17588927838@163.com (X.Z.); duxiyouxiang@163.com (X.D.); fajiefeng@163.com (F.F.); 2 College of Tourism, Xinyang Vocational and Technical College, Xinyang 464000, China; 15236335373@163.com

**Keywords:** *Camellia sinensis*, stem-boring herbivores, rhizosphere microbiome, jasmonic acid cascade, phenylpropanoid biosynthesis

## Abstract

The stem-boring herbivore *Aeolesthes induta* severely threatens tea (*Camellia sinensis*) production, yet the systemic defense mechanisms of tea plants remain largely elusive. The unique age-dependent infestation pattern of *A. induta*—with minimal damage to young tea plants but infestation rates reaching 20% in 30-year-old trees and nearly 100% in ancient tea trees—necessitated field sampling from naturally infested old tea plantations rather than controlled laboratory conditions. By integrating rhizosphere microbiomics, transcriptomics, and multi-omics modeling, this study characterized the defensive regulatory networks of 30-year-old Shuixian tea cultivars under *A. induta* infestation. Rhizosphere microbiome analysis revealed that herbivore attack significantly reshaped the microbial community structure, reducing bacterial diversity and simplifying co-occurrence network complexity. Transcriptomic and metabolomic profiling demonstrated a precise source–sink defense allocation between host tissues. In leaves acting as the photosynthetic source, metabolic reprogramming was dominated by the systematic accumulation of soluble sugars and sugar acids for systemic energy reallocation. Concurrently, the roots acting as the metabolic sink vigorously activated the jasmonic acid (JA) signaling cascade and upregulated genes enriched in phenylpropanoid biosynthesis and alpha-linolenic acid metabolism. This molecular activation drove the substantial de novo synthesis of defensive phenolic acids, while downregulating growth-related flavonoids. Integrated network modeling further highlighted the phenylpropanoid pathway as the central regulatory node coupling transcript–metabolite fluctuations. This study constructs an integrated soil microbiome–root–leaf defense network, providing novel mechanistic insights into plant–borer interactions and a valuable foundation for future insect-resistant breeding programs in the tea industry.

## Simple Summary

The tea plant is an important crop worldwide, but it faces serious threats from pests like the longhorn beetle *Aeolesthes induta*. These beetles bore into the trunks of tea plants, damaging their internal transport system and causing significant losses in tea production. This study investigated how tea plants defend themselves against this hidden attacker. We analyzed the microbes living in the soil around the roots, the chemical changes in the plant’s leaves and roots, and the genes that become active during an attack. We found that infestation changes the community of soil microbes and triggers a coordinated defense strategy in the tea plant. The plant’s leaves, which capture energy from the sun, begin to accumulate sugars, likely to provide energy for defense. At the same time, the roots ramp up production of chemical compounds that can directly fight off the pest, guided by a key plant hormone signal. Our findings reveal that tea plants have a sophisticated way of coordinating defenses between their aboveground and belowground parts. Understanding this natural defense network could help scientists and tea growers develop more sustainable methods to protect tea crops from devastating pests.

## 1. Introduction

The tea plant (*Camellia sinensis*), which yields one of the most widely consumed non-alcoholic beverages globally, has seen a steady expansion in both cultivation area and production, thereby underpinning the economic development of multiple nations [1]. As the native habitat of *C. sinensis* and the world’s leading tea producer, China heavily relies on its tea industry, which occupies a prominent position in the national economy [2]. Nonetheless, as a perennial evergreen crop, the tea plant is vulnerable to a myriad of pests and diseases over its long-term growth cycle [3]. Among these, stem-boring insects, particularly *Aeolesthes induta* Newman (*A. induta*), inflict devastating losses in both tea yield and quality [4,5]. Infestations by *A. induta* are characterized by strong crypticity and exceptional control. The larvae bore exclusively within the trunk, forming intricate galleries that disrupt the vascular system. This disruption severely impedes water and nutrient translocation, compromising tree vigor and ultimately leading to the wilting and mortality of the host plant [6]. Unlike folivorous insects, stem borers damage their hosts in a highly concealed manner, consequently eliciting more complex and distinct defensive pathways [7]. Currently, investigations into tea plant defenses are heavily skewed toward foliage-feeding pests, including the tea geometrid and the tea green leafhopper, *Ectropis grisescens*, *Basilepta melanopus* [8,9,10,11]. Recent multi-omics studies have provided valuable insights into tea plant defense mechanisms against various pests, with integrated metabolomic and transcriptomic analyses revealing key roles for flavonoid and phenylpropanoid pathways in resistance to both pink tea mite and aphid infestations [12,13]. In contrast, the systemic defensive mechanism of *C. sinensis* in response to stem-boring insects remains largely understudied.

Upon insect herbivory, plants initiate defense responses, including the biosynthesis of secondary metabolites and the transcriptional regulation of defense-related genes [14,15]. These responses are not restricted to the localized site of attack; rather, they are transduced throughout the entire plant via systemic signaling, culminating in induced systemic resistance (ISR) [16,17]. Within the phytohormone signaling network, hormones such as jasmonic acid (JA), salicylic acid (SA), and ethylene (ET) play central roles in modulating these defenses [18]. In particular, the JA signaling pathway is widely recognized as a critical defense route against chewing herbivores [19]. Furthermore, secondary metabolites function both directly and indirectly in plant defense. Compounds such as flavonoids, phenylpropanoids, and terpenoids not only directly inhibit pest development but also act as signaling molecules to activate broader defense responses [20,21]. In *C. sinensis*, flavonoids and polyphenols are essential determinants of tea quality and serve as key agents in pest resistance [22]. For instance, by integrating metabolomic and transcriptomic analyses, Chen et al. elucidated the defensive regulatory network in tea plants responding to pink tea mite infestation, revealing a significant enrichment of flavonoid and phenylpropanoid metabolic pathways in resistant cultivars [12]. Similarly, Zhao et al. demonstrated that the JA signaling pathway and flavonoid metabolism are central to the host defense response against the tea green leafhopper [9].

The rhizosphere harbors a highly diverse microbiome that forms intimate associations with plant root systems, playing a pivotal role in promoting plant growth and inducing systemic resistance [23,24]. Studies indicate that when attacked by aboveground herbivores, plants can alter their root exudate profiles to modulate the assembly of the rhizosphere microbial community, effectively issuing a “cry for help” to recruit beneficial microbes for defense [25]. For example, plant growth-promoting microorganisms, such as *Bacillus*, *Pseudomonas*, and *Trichoderma*, can enhance plant insect resistance by triggering ISR [26,27]. In tea plants, the composition of the rhizosphere microbiome is closely correlated with overall host health [28]. Recent research revealed that herbivory by the tea geometrid (*Ectropis grisescens*) significantly alters the structure of the tea plant’s rhizosphere bacterial community, enriching nitrogen-fixing taxa such as *Burkholderia*; re-inoculation with these microbes subsequently bolsters the plant’s insect resistance. Moreover, damage from *E. grisescens* upregulates the expression of genes associated with phenylpropanoid and flavonoid biosynthesis in the roots, suggesting that these specific root exudates may mediate the recruitment of beneficial rhizosphere microbes [10]. However, the specific impact of *A. induta* infestation on the rhizosphere microbiome of tea plants remains largely unexplored.

Recent advances in multi-omics technologies have provided powerful tools for elucidating plant-insect interactions [29,30]. Specifically, transcriptomics reveals global changes in defense-related gene expression, metabolomics captures the dynamic accumulation of defensive metabolites, and microbiomics clarifies the role of the rhizosphere microbiome in host defense [31]. Integrating these datasets allows for the systemic construction of plant defense regulatory networks, thereby providing a theoretical foundation for resistance breeding and sustainable pest management [32,33]. To address the current knowledge gap, the present study investigates the interaction between the tea plant and *A. induta*. By employing a combined transcriptomic, metabolomic, and microbiomic approach, we comprehensively profiled the gene expression and metabolic changes in both leaves and roots following *A. induta* infestation, alongside the structural dynamics of the rhizosphere microbial community. Through this integrated multi-omics analysis, we aim to construct a multi-layered defense network model that couples rhizosphere microecology and root chemical defenses with foliar systemic responses. This proposed model will serve as a foundational framework for further elucidating the synergistic interaction mechanisms underlying tea plant defense against stem-boring pests.

## 2. Materials and Methods

### 2.1. Plant Materials and Experimental Design

**Figure 1:**
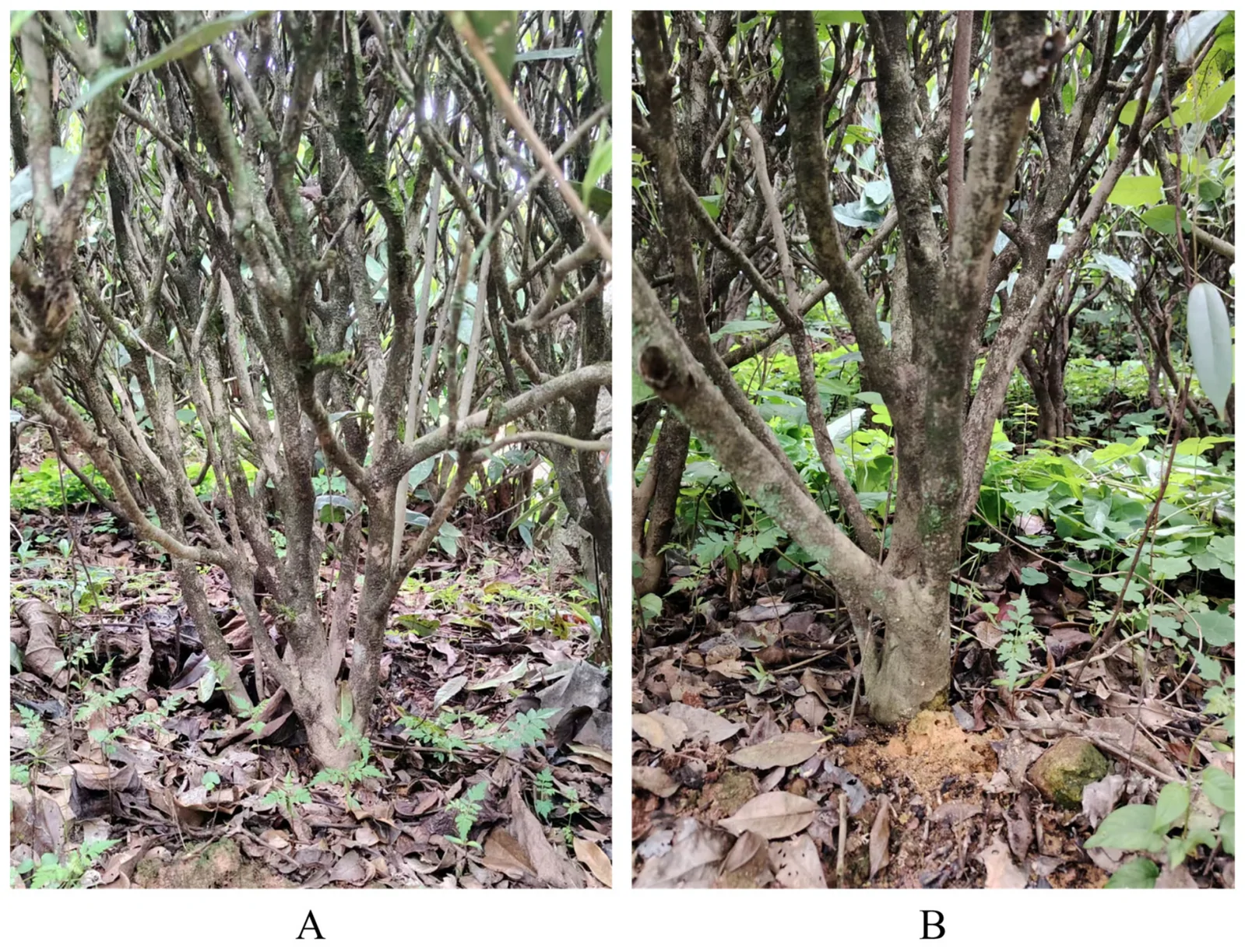
Phenotypes of healthy and *A. induta*-infested tea plants. (**A**) Healthy tea plant; (**B**) Tea plant infested by *A. induta*, where arrows indicate boring holes and frass on the main trunk.

Field experiments were conducted during the peak infestation period of *A. induta* larvae at the Germplasm Repository Nursery of Wuyi University, Wuyishan, Fujian Province, China (118.007887° E, 27.726703° N). Thirty-year-old tea plants (*Camellia sinensis* cv. ‘Shuixian’) exhibiting uniform growth vigor and architecture were selected. The experiment comprised two distinct treatment groups: healthy controls (H) and *A. induta*-infested plants (I). Infested plants were screened and selected based on distinct diagnostic criteria, including adult emergence holes, larval galleries, and fresh frass/wood borings on the main trunks or primary branches (Figure 1). To eliminate microenvironmental bias and root-zone cross-interference, all experimental plants were located within the same nursery block, but the healthy and infested plants were grouped into separate subplots spaced more than 50 m apart. Each treatment group consisted of five independent biological replicates, with each replicate represented by a single distinct plant.

### 2.2. Sample Collection

Rhizosphere soil: After clearing the surface litter under the canopy drip line of each tea plant, rhizosphere soil (0–20 cm depth) was collected at a distance of approximately 20 cm from the main trunk using a sterile soil core sampler. Five cores per plant were pooled into a single biological replicate, passed through a 2 mm mesh sieve to remove gravel and root debris, and immediately stored at −80 °C for subsequent microbial DNA extraction [34].

Roots and Leaves: Fine roots (diameter < 2 mm) corresponding to the sampled rhizosphere zones were excavated, gently rinsed with sterile water to remove adhering soil, and blotted dry [35]. Concurrently, homogeneous sun-exposed new shoots comprising one bud with two leaves were harvested from the upper-middle canopy of each plant. Both root and leaf tissues were immediately snap-frozen in liquid nitrogen and stored at −80 °C for downstream transcriptomic and metabolomic analyses.

### 2.3. Rhizosphere Microbiome Sequencing and Analysis

Total genomic DNA from the rhizosphere soil was extracted using the PowerSoil^®^ DNA Isolation Kit (Mo Bio Laboratories, Carlsbad, CA, USA) [36]. DNA integrity and concentration were verified via 1% agarose gel electrophoresis and a NanoDrop 2000 spectrophotometer (Thermo Fisher Scientific, Waltham, MA, USA). The V3–V4 hypervariable regions of the bacterial 16S rRNA gene were amplified using primers 338F (5′-ACTCCTACGGGAGGCAGCAG-3′) and 806R (5′-GGACTACHVGGGTWTCTAAT-3′), and the fungal ITS1 region was amplified using primers ITS1F (5′-CTTGGTCATTTAGAGGAAGTAA-3′) and ITS2R (5′-GCTGCGTTCTTCATCGATGC-3′). PCR amplification was performed with an annealing temperature of 56 °C for bacteria and 55 °C for fungi [37]. The resulting PCR products were purified using Agencourt AMPure XP beads (Beckman Coulter, Brea, CA, USA), quantified via a Qubit fluorometer (Thermo Fisher Scientific, Waltham, MA, USA), and subjected to paired-end sequencing (PE250) on the Illumina NovaSeq 6000 platform (Illumina, San Diego, CA, USA).

Raw sequencing reads were quality-filtered and denoised to generate amplicon sequence variants (ASVs) using the DADA2 plugin within the QIIME 2 platform (version 2024.5) [38]. Taxonomic assignment of representative ASVs was performed against the Silva 138.1 (bacteria) and UNITE 8.0 (fungi) databases. Alpha diversity metrics (Chao1 and Shannon indices) and beta diversity (Principal Coordinate Analysis, PCoA, based on Bray–Curtis distance) were calculated [39]. Core discriminatory microbial taxa between groups were identified using Random Forest modeling, and microbial co-occurrence networks were constructed using the igraph package (version 1.3.5) in R (version 4.2.0) [40].

### 2.4. Untargeted Metabolomics Analysis

Frozen tissue samples (100 mg) were ground in liquid nitrogen and extracted with 1.0 mL of 70% aqueous methanol containing an internal standard [41]. Following centrifugation (12,000 rpm, 4 °C, 10 min), the supernatant was filtered through a 0.22 μm membrane for analysis. Extracts were profiled using a UHPLC-MS/MS system consisting of a Vanquish UHPLC system (Thermo Fisher Scientific, Bremen, Germany) coupled with a Thermo Scientific Q Exactive™ HF hybrid quadrupole-Orbitrap mass spectrometer (Thermo Fisher Scientific, Bremen, Germany), equipped with a Hypersil Gold column (1.9 μm, 2.1 × 100 mm; Thermo Fisher Scientific, Waltham, MA, USA) operating in both ESI(+) and ESI(−) modes. Peak extraction, alignment, and normalization were performed using Progenesis QI software (version 3.0), and metabolites were identified against the METLIN (https://metlin.scripps.edu) and HMDB databases (https://hmdb.ca) [42]. Differentially accumulated metabolites (DAMs) were screened based on thresholds of variable importance in projection (VIP) ≥ 1, |log2 (Fold Change)| ≥ 1, and an adjusted *p*-value < 0.05 from a two-tailed Student’s *t*-test [43]. Orthogonal partial least squares-discriminant analysis (OPLS-DA) and KEGG pathway enrichment analyses were subsequently performed.

### 2.5. Transcriptomics Analysis

Total RNA was extracted using the RNAprep Pure Plant Kit (TIANGEN, Beijing, China) [44], and its quality was verified using an Agilent 2100 Bioanalyzer (Agilent Technologies, Santa, IN, USA). Strand-specific transcriptomic libraries were constructed using the NEBNext^®^ Ultra™ RNA Library Prep Kit (New England Biolabs, lpswich, MA, USA) and sequenced on the Illumina NovaSeq 6000 platform (Illumina, San Diego, CA, USA) in PE150 mode [45]. Clean reads were obtained by removing adapters and low-quality data, followed by de novo transcriptome assembly into reference unigenes using Trinity (version 2.8.5) [46]. Unigene expression levels were quantified as FPKM values using RSEM (version 1.3.1) [47]. Differentially expressed genes (DEGs) between treatments were identified using the DESeq2 package (version 1.36.0) with thresholds of |log2 (Fold Change)| ≥ 1 and an adjusted *p*-value < 0.05 [48]. Functional annotation and pathway enrichment of the DEGs were performed using Blast2GO (version 5.2) and KOBAS (version 3.0) against the GO (version 2024-11-03; https://geneontology.org) and KEGG databases (Release 119.0+, August 2026; PATHWAY and BRITE databases updated 2026/07/24; https://www.kegg.jp) [49,50]. Weighted gene co-expression network analysis (WGCNA) was executed in R (version 4.2.0) using the WGCNA package (version 1.71) to identify highly co-expressed functional gene modules correlated with *A. induta* infestation [51,52]. Soft-thresholding power was selected based on scale-free topology fit (R^2^ > 0.8), with a minimum module size of 30 genes and a module merge cut height of 0.25.

### 2.6. Quantitative Real-Time PCR (qRT-PCR) Verification

To validate the transcriptomic data, six key DEGs involved in jasmonic acid biosynthesis and phenylpropanoid metabolism in roots, including phenylalanine ammonia-lyase-like (*PAL*), 4-coumarate-CoA ligase (*4CL*), caffeoyl-CoA O-methyltransferase-like(*COMT*), peroxidase 5-like (*POD5*), and trans-cinnamate 4-monooxygenase-like (*C4H*), were quantified via qRT-PCR. Assays were conducted on a LightCycler^®^ 480 system using the SYBR^®^ Premix Ex Taq™ II Kit (TaKaRa, Kyoto, Japan). GAPDH was employed as the internal reference standard, and relative expression levels were calculated via the 2^−ΔΔCT^ method. The experiment included three biological replicates (each derived from an individual plant) and three technical replicates per biological replicate [53]. Gene-specific primer sequences designed by Primer Premier 5.0 are listed in Table S1.

### 2.7. Targeted Quantitative Metabolomic Analysis

For absolute quantification of JA, ferulic acid, and p-coumaric acid, frozen root samples were homogenized and extracted via ultrasonic-assisted extraction using ice-cold 80% methanol. Extracts were analyzed under the multiple reaction monitoring (MRM) mode via UPLC-MS/MS using authentic standards (Sigma-Aldrich, St. Louis, MO, USA). Chromatographic separation conditions mirrored those described in Section 2.4. Absolute concentrations (μg/g, fresh weight) were determined using standard calibration curves constructed from multi-point standard solutions across five biological replicates.

### 2.8. Statistical Analysis

Statistical analysis was executed using R (v4.2.0) and SPSS version 26.0. Pairwise comparisons between the healthy control (H) and infested (I) groups were evaluated using a two-tailed Student’s *t*-test (for normally distributed data) or the Wilcoxon rank-sum test (for non-normally distributed data). Data visualization was performed using ggplot2, pheatmap, and GraphPad Prism 8.0. Statistical significance thresholds were established at *p* < 0.05 (*) and *p* < 0.01 (**). All quantitative values are presented as the mean ± standard deviation (SD).

## 3. Results

### 3.1. Impact of A. induta Infestation on Rhizosphere Microbial Community Structure

**Figure 2:**
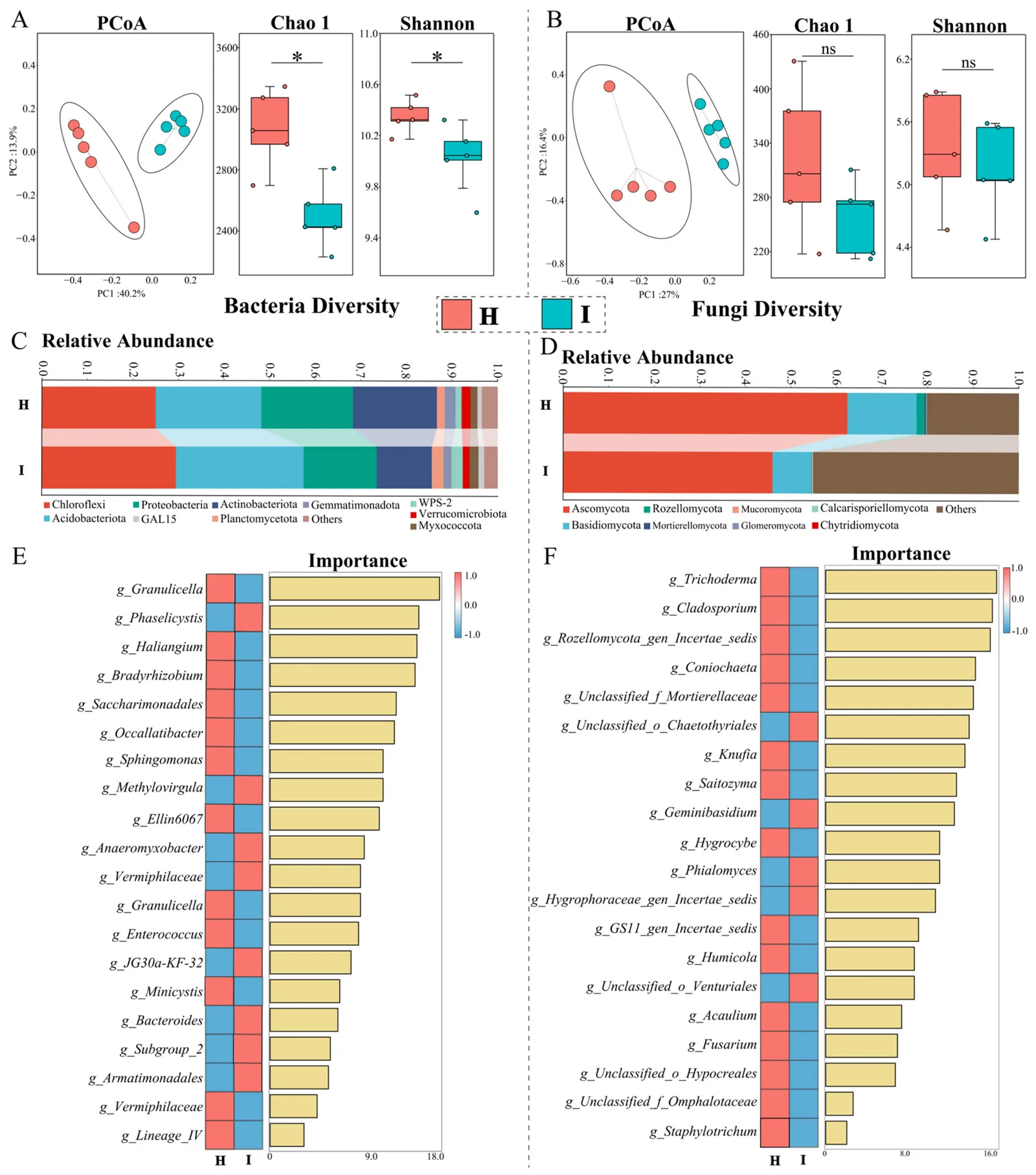
Effects of *A. induta* infestation on the community structure of the rhizosphere soil microbiome. (**A**,**B**) Principal Coordinate Analysis (PCoA) of bacterial and fungal communities based on Bray–Curtis distances, with inset box plots representing the alpha diversity metrics, Chao1 and Shannon indices, where * indicates *p* < 0.05 and ns indicates no significant difference. (**C**,**D**) Relative abundance profiles of the top 10 bacterial and fungal phyla. (**E**,**F**) Random forest analysis of bacterial and fungal genera, where the plots on the left exhibit the top 20 genera ranked by their variable importance in group differentiation, and the heatmaps on the right illustrate the relative abundance distribution of these corresponding genera across the healthy (H) and infested (I) groups.

High-throughput sequencing analysis revealed that stem-boring by *A. induta* significantly altered the balance of the rhizosphere microecology. Principal Coordinate Analysis (PCoA) demonstrated a clear and significant separation in both bacterial and fungal community structures between the healthy control (H) and infested (I) tea plants, with PC1 explaining 40.2% of the total variance in the bacterial community (Figure 2A,B). This community divergence was accompanied by a significant decline in ecological diversity. Specifically, herbivore infestation significantly suppressed rhizosphere bacterial richness (with the Chao1 index decreasing from 3149 to 2590) and diversity (Shannon index), whereas the alpha diversity of the fungal community remained statistically stable (Figure 2A,B).

At the taxonomic level, *A. induta* infestation induced a pronounced compositional shift in the rhizosphere bacterial community (Figure 2C). Random Forest modeling was further employed to identify the key indicator taxa (biomarkers) driving these inter-group differences. In the healthy rhizosphere, taxa associated with critical ecological functions such as the nitrogen-cycling genus *Bradyrhizobium* and the plant growth-promoting genus *Sphingomonas* exhibited a substantial decrease in their variable importance scores following infestation. Conversely, *Phaselicystis* and *Methylovirgula*, which are typically associated with oligotrophic conditions, stress tolerance, and complex carbon degradation, emerged as the top contributors to group discrimination in the infested rhizosphere (Figure 2E).

**Figure 3:**
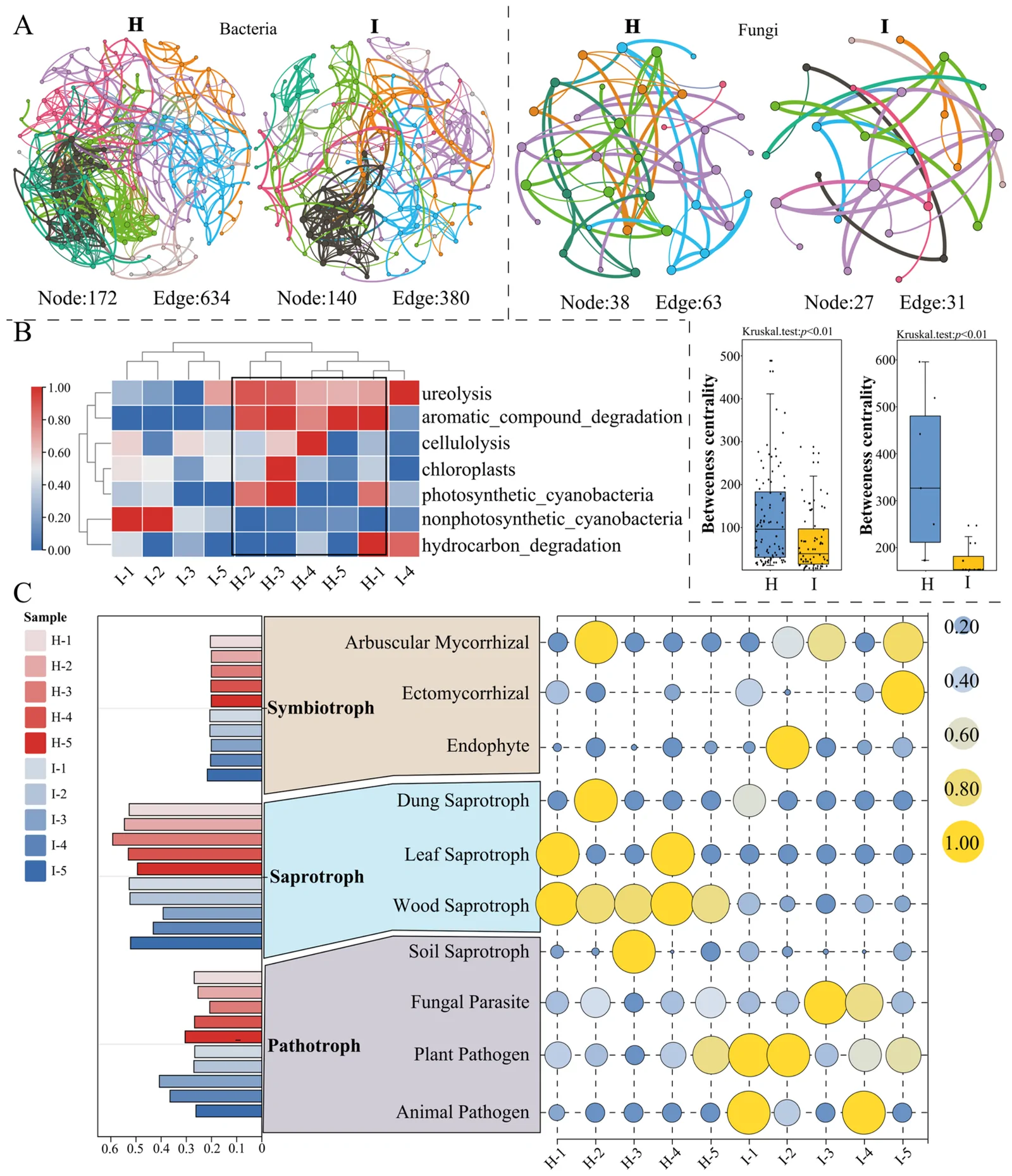
Effects of *A. induta* infestation on the co-occurrence networks and functional profiles of the rhizosphere microbiome. (**A**) Molecular ecological networks of bacterial and fungal communities in the healthy (H) and infested (I) groups, where nodes represent amplicon sequence variants (ASVs), and edges represent statistically significant correlations (|r| > 0.6, *p* < 0.05) between ASVs. (**B**) Heatmap of predicted bacterial functions based on FAPROTAX and box plots of network betweenness centrality, where indicates *p* < 0.01. (**C**) Bubble plot illustrating fungal trophic modes and specific functional guilds predicted by FUNGuild.

This structural transition further led to the simplification and functional degradation of the microbial co-occurrence networks. Network analysis indicated that *A. induta* infestation caused the number of nodes and edges in the bacterial network to decrease sharply from 172 and 634 to 140 and 380, respectively, reflecting a weakening of both synergistic and antagonistic interactions within the community (Figure 3A). Furthermore, the betweenness centrality, which characterizes the importance of nodes in information transfer, decreased significantly, indicating that key interactions within the microbial community were disrupted.

Functional prediction analysis corroborated these structural changes. In the infested group (I), the abundance of metabolic functional genes associated with ureolysis and aromatic compound degradation decreased significantly, whereas genes linked to environmental stress responses, such as non-photosynthetic cyanobacterial groups, were significantly enriched (Figure 3B). For the fungal community, functional prediction showed that saprotrophic fungi were progressively replaced by pathogenic and symbiotic guilds post-infestation (Figure 3C). This shift indicates that *A. induta* damage not only disrupts the basal metabolic network of the rhizosphere but also increases the ecological risk of secondary pathogen infestation in tea plants. Collectively, these findings demonstrate a distinct pattern: instead of recruiting specific beneficial microbes, *A. induta* infestation establishes a simplified, functionally degraded rhizosphere microecological equilibrium dominated by stress-tolerant taxa.

### 3.2. Metabolic Reprogramming in Tea Leaves and Roots Induced by A. induta Infestation

**Figure 4:**
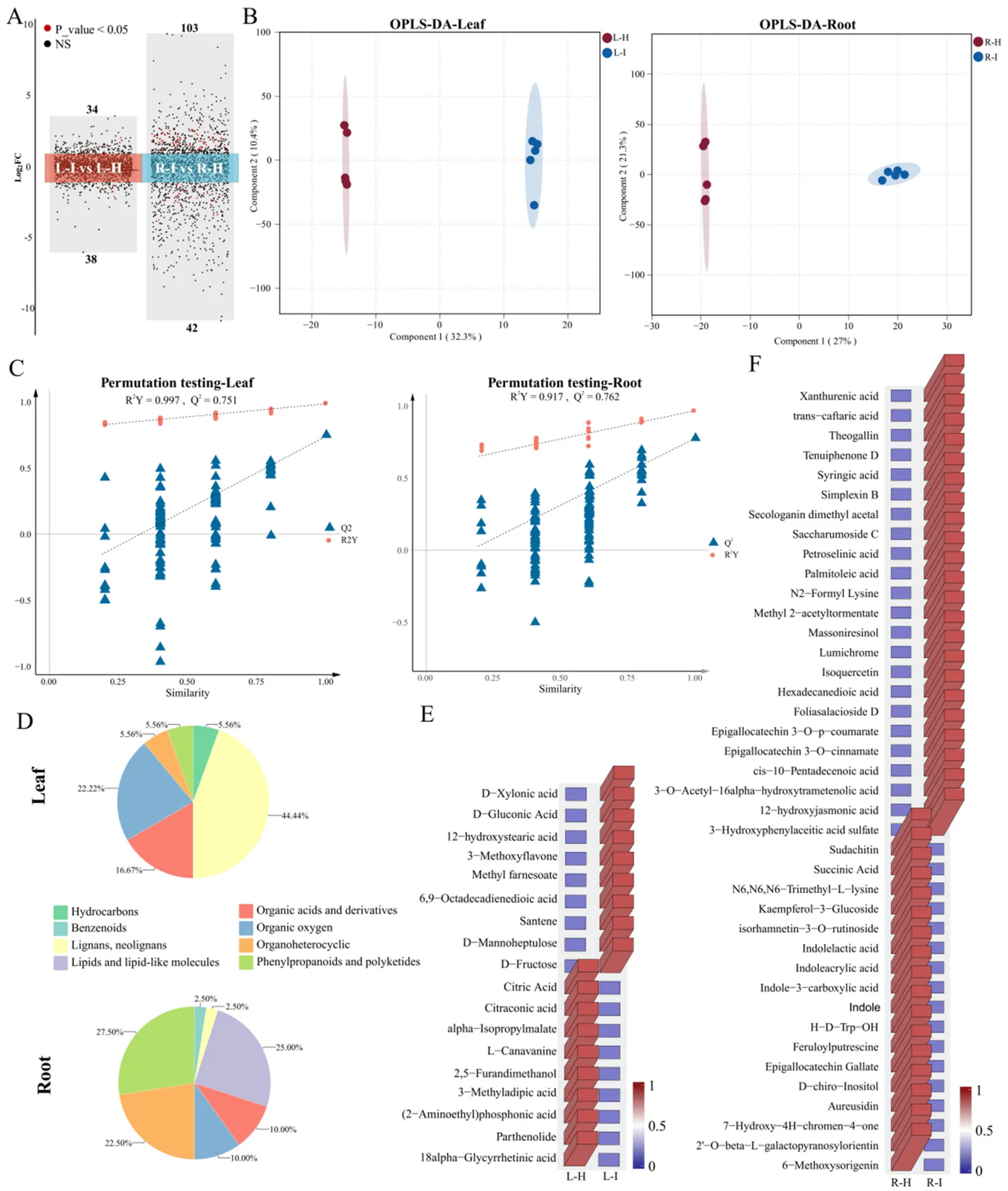
Effects of *A. induta* infestation on the metabolic profiles of tea plant leaves and roots. (**A**) Volcano plots of differentially expressed metabolites (DEMs) in leaves (L) and roots (R), with the screening threshold defined as |log2FC| ≥ 1 and *p* < 0.05. (**B**) Score plots of the OPLS-DA models for leaves and roots. (**C**) Permutation tests (*n* = 200) of the OPLS-DA models, where R^2^Y indicates the goodness-of-fit and Q^2^ indicates the predictability of the model. (**D**) Pie charts illustrating the chemical classification profiles of the identified DEMs. (**E**) Heatmaps exhibiting the relative abundance of the top 20 DEMs ranked by their contribution in leaves and (**F**) roots.

Untargeted metabolomic analysis revealed that tea plants deployed a highly tissue-specific defense strategy in response to *A. induta* infestation. Volcano plot analysis demonstrated that the magnitude of the metabolic response in roots far exceeded that in leaves; a total of 145 significantly differentially expressed metabolites (103 upregulated) were identified in the roots, compared to only 72 in the leaves (Figure 4A). Orthogonal Projections to Latent Structures-Discriminant Analysis (OPLS-DA) models confirmed distinct metabolic profiles between the control and infested groups for both leaves and roots, with the models exhibiting high explanatory and predictive capabilities (Figure 4B,C).

The metabolic strategy of leaves was characterized by a reallocation of energy resources. In the leaves of infested plants, defense-related terpenoids (such as 18α-glycyrrhetinic acid and parthenolide) and the defensive non-protein amino acid L-canavanine exhibited a decreasing trend. Concurrently, soluble sugars, including D-fructose and D-mannoheptulose, as well as sugar acids, such as D-xylic acid and D-gluconic acid, accumulated significantly (Figure 4E). This concentrated enrichment of carbohydrate metabolites suggests that, under systemic signaling, leaves actively reduce investment in local secondary defenses. Instead, they rewire carbohydrate metabolism to provide energy support or signal transduction for whole-plant systemic defense and the intensive response in roots.

Conversely, the roots prioritized a direct chemical defense mediated by the JA signaling pathway. Functional classification of the significantly differential metabolites showed that the roots were enriched with a substantial number of phenylpropanoids, polyketides, and lipid derivatives (Figure 4D). Notably, the defense signaling molecule 12-hydroxyjasmonic acid was markedly upregulated in infested roots, providing clear evidence of the precise activation of the JA-mediated defense pathway in the belowground tissues (Figure 4F). Driven by JA signaling, the roots synthesized and accumulated large amounts of phenolic acids and aromatic compounds with potential insecticidal activity, such as theogallin, syringic acid, and phenolic acid esters (e.g., epigallocatechin 3-O-p-coumarate/cinnamate). In contrast, flavonoids and their glycosides (such as isorhamnetin and kaempferitrin), as well as indole biosynthetic precursors, which were initially abundant in healthy roots, were downregulated (Figure 4F). This pronounced shift in the metabolic profile indicates that, under the severe physical wounding and chemical elicitation caused by *A. induta* boring, roots rapidly transition from basal metabolism for growth and development to a defense-dominated metabolic mode guided by JA signaling and weaponized by phenolic acids.

Integrating the metabolomic datasets highlights a coordinated and comprehensive defense allocation between the source and sink organs of the tea plant. In leaves acting as the photosynthetic source, metabolic reprogramming is characterized by the substantial accumulation of soluble sugars and sugar acids alongside the downregulation of local secondary defenses. This accumulation not only mobilizes energetic resources and potential signaling molecules for systemic defense but also serves a vital role in osmotic adjustment. Meanwhile, the roots acting as the metabolic “sink” at the direct site of infestation precisely activate the JA signaling cascade to channel incoming carbon skeletons into the phenylpropanoid and aromatic pathways. This signaling shift actively promotes the synthesis of defensive phenolic acids, theogallin, and phenolic acid esters while suppressing the biosynthetic precursors of growth-related flavonoids. This coordinated transition from foliar energy reallocation to root chemical defense reinforcement constitutes the core systemic defense strategy of tea plants against stem-boring herbivores.

### 3.3. Transcriptomic Reprogramming in Tea Leaves and Roots Induced by A. induta Infestation

**Figure 5:**
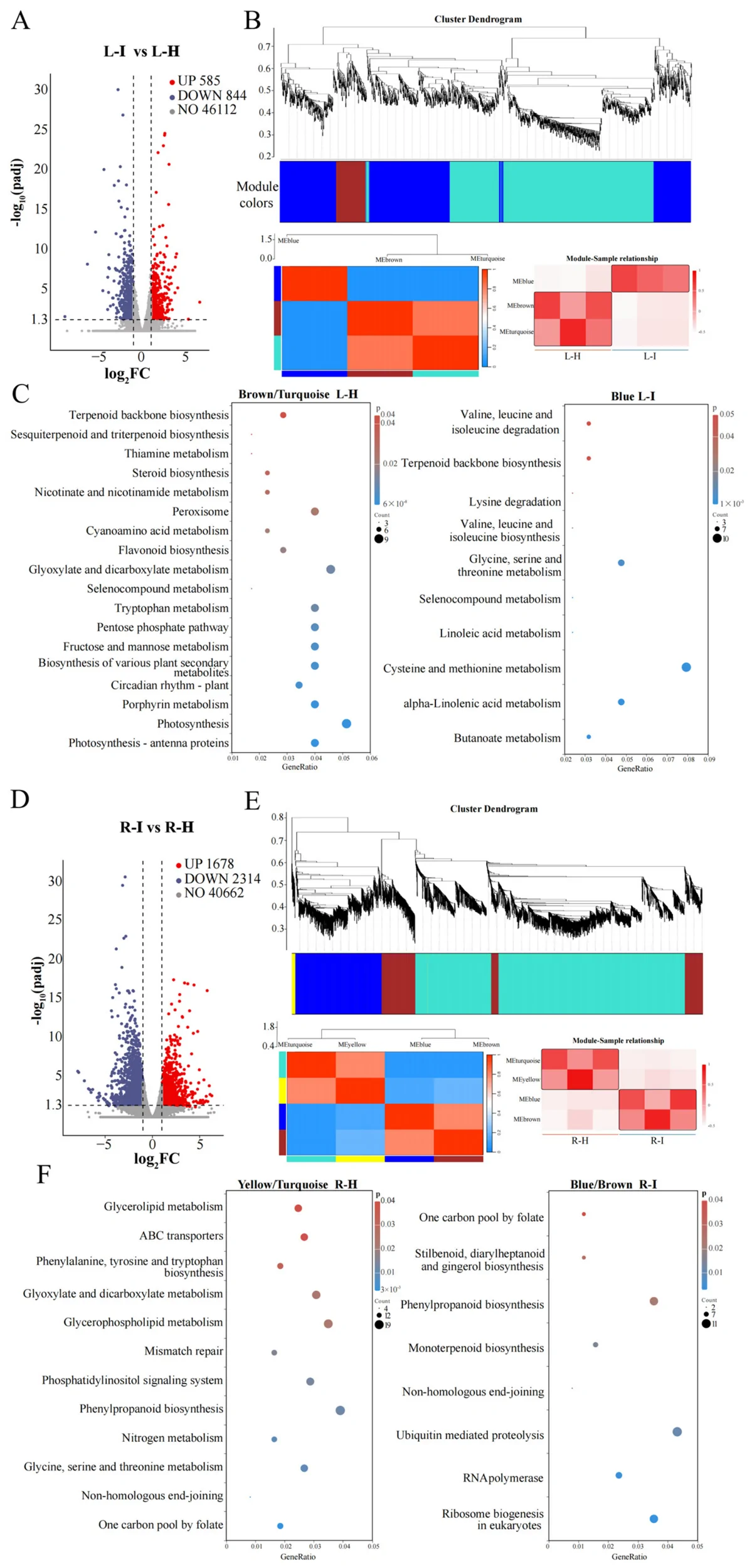
Effects of *A. induta* infestation on the transcriptome of tea plant leaves and roots, alongside WGCNA profiles. (**A**) Volcano plots of differentially expressed genes (DEGs) in leaves and (**D**) roots, with the screening threshold defined as |log2FC| ≥ 1 and padj < 0.05. (**B**) Heatmaps showing the correlation between WGCNA modules and treatment groups in leaves and (**E**) roots, with the numbers inside the cells representing the correlation coefficients and the values in parentheses indicating the statistical significance (*p*-values). (**C**) KEGG pathway enrichment analysis of the foliar blue module and (**F**) the root blue and brown modules, where the bubble size corresponds to the number of assigned genes, and the color intensity dictates the significance of enrichment.

To explore the molecular basis underlying the observed metabolic alterations, we conducted transcriptomic sequencing on both leaves and roots. Volcano plot analysis identified 585 upregulated and 844 downregulated differentially expressed genes (DEGs) in the leaves under the L-I versus L-H comparison (Figure 5A). In contrast, the number of DEGs increased substantially in the roots under the R-I versus R-H comparison, reaching 1678 upregulated and 2314 downregulated genes (Figure 5D). This divergence indicates a more robust transcriptional response in the roots, which represent the primary site of herbivore infestation, than in the distal leaves. Weighted gene co-expression network analysis (WGCNA) was subsequently employed to partition the complex DEGs into highly coordinated expression modules, thereby elucidating the core tissue-specific defense strategies deployed against *A. induta* (Figure 5B,E).

In the leaves, the brown and turquoise modules displayed high positive correlations with the healthy control (H) status. Hub genes within these modules were primarily enriched in pathways related to light harvesting, including photosynthesis-antenna proteins, photosynthesis, carbon fixation in photosynthetic organisms (Calvin cycle), and the pentose phosphate pathway. Concurrently, basal physiological processes such as circadian rhythm and protein processing in the endoplasmic reticulum were actively maintained. However, following *A. induta* infestation, the leaf transcriptomic profile shifted substantially, marked by the significant activation of the blue module, which was highly correlated with the infestation (I) status. This module was significantly enriched in pathways associated with defense signaling and secondary metabolism, including alpha-linolenic acid metabolism (the precursor pathway for JA biosynthesis), linoleic acid metabolism, phenylpropanoid biosynthesis, and flavonoid biosynthesis. Additionally, the enrichment of amino acid metabolic pathways, such as lysine degradation, suggested a reallocation of nitrogen resources under stress. These findings demonstrate that, despite the lack of direct foliar feeding, systemic defense signals prompt leaves to downregulate basal photosynthesis and energy metabolism, thereby redirecting metabolic flux toward lipid signaling and secondary metabolite biosynthesis (Figure 5C).

As the primary defense hub responding to *A. induta* attack, the roots exhibited a more complex reorganization of their gene co-expression network. Under the healthy control (H) status, the yellow and turquoise modules maintained basal root homeostasis, showing significant enrichment in glycerolipid metabolism, the phosphatidylinositol signaling system for membrane structure and transmembrane signaling, ABC transporters, and mismatch repair. This enrichment reflects a coordinated equilibrium of lipid metabolism, signal transduction, and genomic stability in healthy roots. Conversely, the blue and brown modules, which were highly correlated with the infestation (I) status, revealed an intensive deployment of defenses in the roots. At the chemical defense level, biosynthetic pathways for phenylpropanoids, stilbenoids, diarylheptanoids, gingerol, and monoterpenoids were markedly upregulated, indicating the de novo synthesis of phytoalexins and volatile organic compounds with potential toxic or deterrent properties. To sustain this high-intensity secondary metabolism and defense response, significant resource reallocation and compensatory mechanisms occurred within the roots. On the one hand, ubiquitin-mediated proteolysis was significantly enriched, suggesting accelerated degradation of damaged proteins to provide amino acid substrates for the synthesis of new defense-related proteins. On the other hand, the marked upregulation of genes involved in RNA polymerase and ribosome biogenesis in eukaryotes reflected a profound reprogramming of the transcriptional and translational machinery under herbivore stress. The coordinated activation of these molecular processes establishes an efficient defense and metabolic compensation system in tea roots against stem-boring pests (Figure 5F).

To identify the critical regulatory nodes within the root chemical defense network, we focused on the blue module that exhibited a strong positive correlation with the infestation treatment. By calculating the module membership (kME) values, we identified the top 20 hub genes with the highest connectivity. Functional annotation revealed that these hub genes primarily encode key enzymes in the JA biosynthetic pathway and rate-limiting enzymes in phenylpropanoid metabolism, suggesting that these genes may serve as potential molecular switches regulating defensive metabolic flux in the roots. Among these hub genes, the JA pathway genes include *LOX* (lipoxygenase), *AOS* (allene oxide synthase), and *OPR* (12-oxophytodienoate reductase), which catalyze sequential reactions from linolenic acid to JA. The phenylpropanoid pathway genes include *PAL* (phenylalanine ammonia-lyase), *C4H* (cinnamate 4-hydroxylase), *4CL* (4-coumarate-CoA ligase), and *COMT* (caffeoyl-CoA O-methyltransferase), which function in the conversion of phenylalanine to phenolic acid precursors and lignin monomers. Additionally, several MYB and WRKY family transcription factors were identified among the hub genes, which are known to regulate phenylpropanoid gene expression by binding to specific promoter elements. This co-expression pattern suggests that the blue module represents a coordinated transcriptional program linking JA signaling to the downstream phenylpropanoid pathway, with transcription factors potentially orchestrating the defense metabolic flux in infested roots.

### 3.4. Integrated Transcriptomic and Metabolomic Analysis Revealing the Regulatory Defense Network

To elucidate the coordinated response mechanisms between the transcriptional and metabolic layers in tea plants, we conducted an integrated transcriptomic and metabolomic analysis for both leaves and roots. By constructing an interactive KEGG pathway-gene–metabolite correlation network combined with Pearson correlation analysis, we successfully decoded the regulatory linkages between gene expression and metabolic fluctuations.

**Figure 6:**
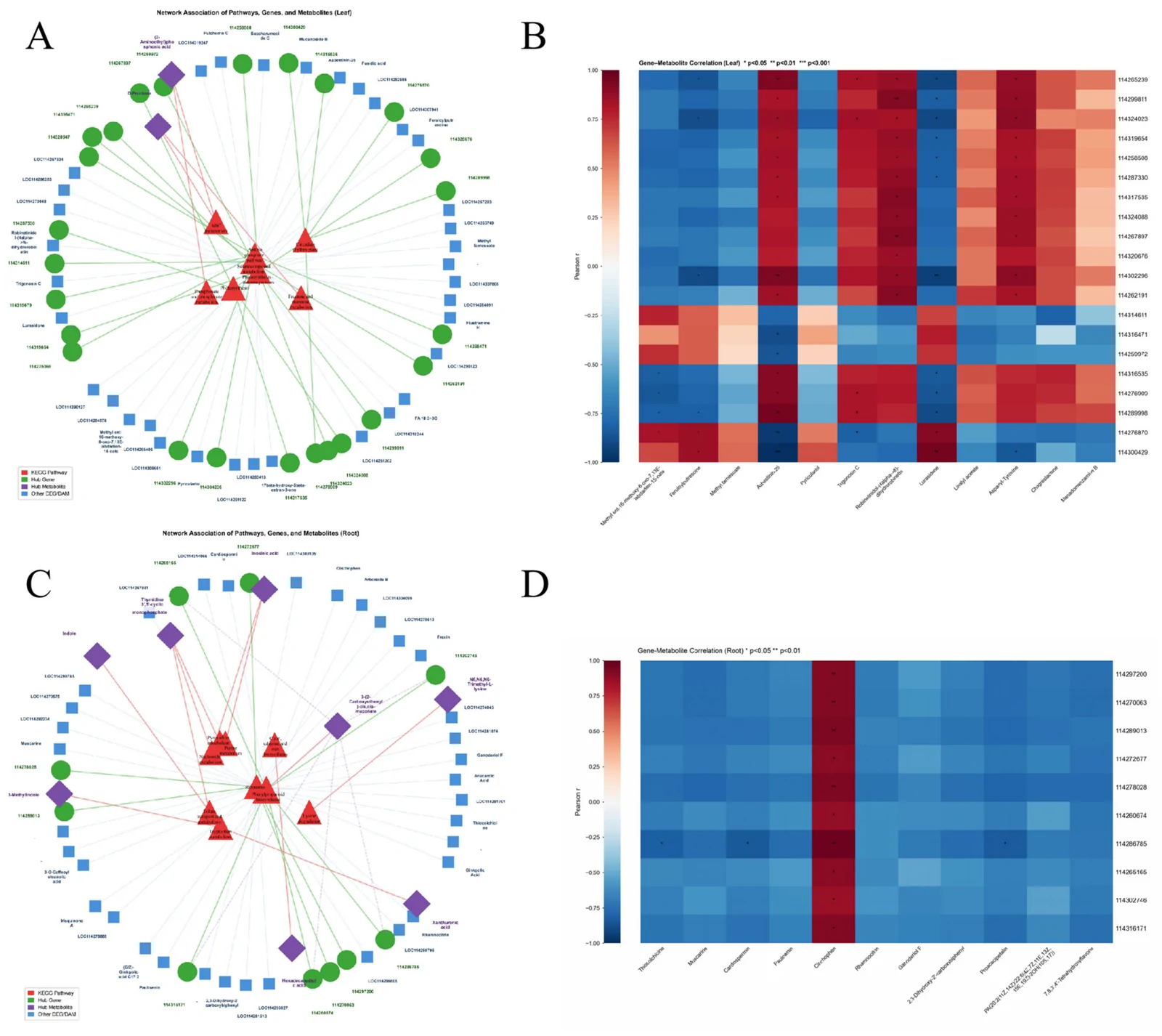
Integrated transcriptomic–metabolomic analysis revealing the defense regulatory networks. (**A**) Interaction network connecting KEGG pathways, hub genes, and hub metabolites in leaves, where red triangular nodes represent KEGG pathways, green circular nodes represent hub genes, and purple diamond nodes represent hub metabolites, with purple dashed edges indicating gene–metabolite interactions. (**B**) Heatmap illustrating Pearson correlation coefficients between foliar genes and metabolites, with red and blue indicating positive and negative correlations, respectively, and asterisks denoting statistically significant pairs (*p* < 0.05). (**C**) Interaction network connecting KEGG pathways, hub genes, and hub metabolites in roots, with the same node and edge definitions as in (**A**). (**D**) Heatmap illustrating Pearson correlation coefficients between root genes and metabolites, with the same color and significance notations as in (**B**).

At the leaf level, the association network diagram (Figure 6A) showed that the hub genes (represented as green nodes) and hub metabolites (represented as purple nodes) were tightly distributed around core pathway nodes. The gene–metabolite interactive edges, denoted by purple dashed lines, revealed multiple closely coupled transcript–metabolite regulatory relationships. Pearson correlation analysis (Figure 6B) further identified several statistically significant gene–metabolite regulatory pairs (*p* < 0.05). Specifically, genes involved in carbohydrate metabolism, such as those encoding hexokinase and fructokinase, exhibited strong positive correlations (r > 0.8) with the accumulation of soluble sugars and sugar acids, including D-fructose and D-gluconic acid. This correlation provides direct molecular evidence supporting the foliar energy reallocation strategy.

At the root level, the association network diagram (Figure 6C) demonstrated that the phenylpropanoid biosynthesis pathway served as the central hub node in the root response to *A. induta* infestation. Multiple hub genes, including phenylalanine ammonia-lyase (*PAL*), cinnamate 4-hydroxylase (*C4H*), and 4-coumarate-CoA ligase (*4CL*), along with hub metabolites such as ferulic acid, p-coumaric acid, caffeic acid, and various phenolic acid esters, were highly correlated with this pathway, confirming its predominant role in belowground chemical defense. Pearson correlation analysis (Figure 6D) identified several strong positive correlations (r > 0.8) in the roots, particularly the high connectivity between the *PAL* gene and ferulic acid, as well as between *C4H* and p-coumaric acid. These high coefficients strongly suggest that these genes directly modulate the biosynthesis of their respective metabolites. Furthermore, genes associated with JA biosynthesis displayed significant positive correlations with 12-hydroxyjasmonic acid, further validating the central role of the JA signaling pathway in driving root defenses.

In summary, the integrated multi-omics analysis provides robust molecular evidence for a coordinated source-to-sink defense model, where foliar energetic resources are systematically reallocated to reinforce belowground chemical defenses, providing multiple critical regulatory pairs as prospective targets for subsequent functional validation.

### 3.5. Validation of Key Differentially Expressed Genes and Metabolites

**Figure 7:**
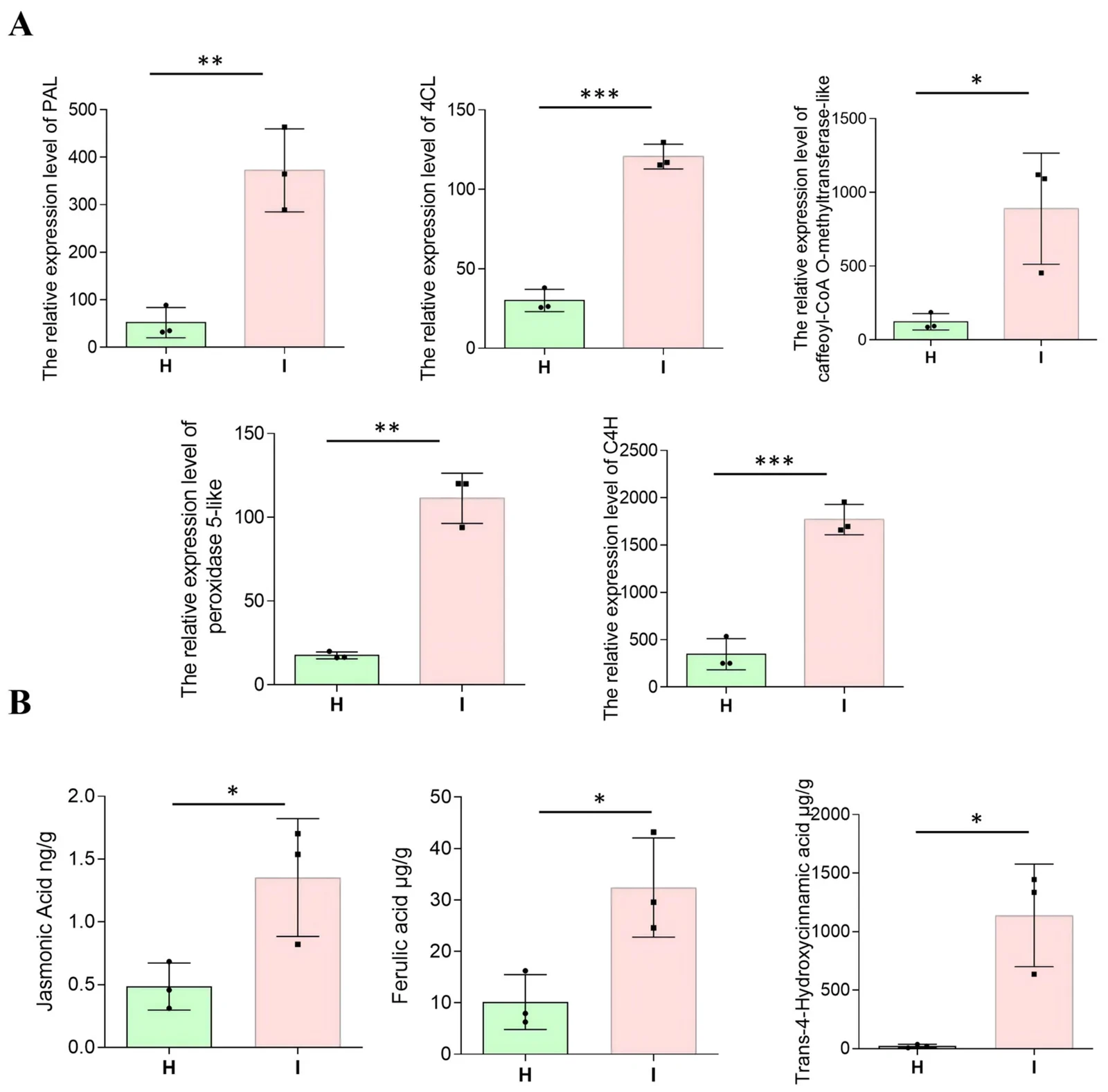
Validation of key differentially expressed genes and metabolites. (**A**) qRT-PCR validation of the relative expression levels of five key genes in the roots under healthy (H) and infested (I) treatments. (**B**) Targeted quantitative metabolomic analysis showing the absolute concentrations of JA, ferulic acid, and p-coumaric acid under healthy (H) and infested (I) treatments, where asterisks indicate statistical significance at * *p* < 0.05, ** *p* < 0.01, and *** *p* < 0.001.

To verify the reliability of the transcriptomic dataset, six key differentially expressed genes (DEGs) in the roots were selected for quantitative real-time PCR (qRT-PCR) analysis. Compared with the healthy control (H), *A. induta* infestation (I) significantly upregulated the expression levels of phenylalanine ammonia-lyase-like (*PAL*), 4-coumarate-CoA ligase (*4CL*), caffeoyl-CoA O-methyltransferase-like (*COMT*), peroxidase 5-like (*POD5*), and trans-cinnamate 4-monooxygenase-like (*C4H*), with fold-changes of 7.21, 4.02, 7.27, 6.35, 4.50, and 5.10, respectively (Figure 7A). This expression trend demonstrated high consistency with the RNA-sequencing data (Pearson r = 0.94), thereby confirming the technical reliability of the transcriptome analysis.

Targeted quantitative metabolomic analysis showed that the concentration of JA in the roots under the I treatment was significantly higher than that in the H treatment, increasing from 0.49 to 1.35 ng/g. Concurrently, downstream products of the phenylpropanoid biosynthetic pathway also exhibited substantial accumulation, with ferulic acid increasing from 10.15 μg/g to 32.42 μg/g, and p-coumaric acid increasing from 455.80 μg/g to 1472.56 μg/g (Figure 7B). These quantitative results closely match the upregulation trends observed for 12-hydroxyjasmonic acid and various phenolic acids in the untargeted metabolomics, providing absolute quantitative confirmation of JA signaling activation and phenylpropanoid metabolism enhancement in infested tea roots.

## 4. Discussion

### 4.1. Response and Feedback of the Rhizosphere Microbiome to A. induta Infestation

Contrary to the classical ‘cry-for-help’ defense paradigm, *A. induta* infestation did not enrich typical PGPR, such as *Bacillus* or *Pseudomonas*; instead, the rhizosphere shifted toward oligotrophic taxa [26]. Instead, the rhizosphere shifts toward an enrichment of *Acidobacteriota* and *Chloroflexi*, which are generally categorized as slow-growing oligotrophs proficient in utilizing recalcitrant carbon sources. Integrating these findings with the observed simplification of the co-occurrence network and the diminished importance of core genera, such as *Bradyrhizobium*, we hypothesize that this community succession may reflect a passive niche-filtering process driven by alterations in root exudate compositions following stem-boring damage [26]. This shift contrasts with the active defense strategy proposed by the “cry-for-help” hypothesis, which posits that plants actively recruit beneficial microbes under herbivore attack [25]. Although this new equilibrium dominated by stress-tolerant taxa may facilitate the decomposition of specific root necrotic debris or secreted defensive compounds, its long-term feedback on overall host fitness likely imposes severe ecological costs, particularly in compromising the cooperative resistance against phytopathogens. This potential downside is further supported by the functional prediction, which shows an increased proportion of pathotrophic guilds within the fungal community. Future investigations utilizing the isolation, cultivation, and back-inoculation of these key bacterial strains are required to experimentally validate their definitive ecological functions.

It is important to note that the microbiome shifts observed in this study are correlative in nature. Whether the altered rhizosphere community is a consequence of the plant’s defense response (e.g., due to changes in root exudate composition) or a contributing factor that modulates defense intensity remains unresolved. Nevertheless, the synchronous activation of root chemical defenses and the concurrent simplification of microbial network complexity suggest that the plant’s metabolic reprogramming is the primary driver, with the microbiome responding passively to the altered belowground environment [25,26]. This interpretation is consistent with the observed enrichment of stress-tolerant oligotrophic taxa rather than classic PGPR, which would be expected if active recruitment were occurring. Future studies employing root exudate transplantation or synthetic microbial community reconstitution are required to dissect the causal relationships between these concurrent events.

### 4.2. JA Signaling as the Central Regulator of Root Defense Responses

The integrated metabolomic and transcriptomic datasets from this study strongly indicate that the JA signaling pathway serves as the central regulator of the root defense response. The prominent accumulation of 12-hydroxyjasmonic acid in the roots, alongside the synchronous upregulation of genes associated with JA biosynthesis (*LOX*, *AOS*) and signal transduction within the blue module, demonstrates that *A. induta* infestation effectively activates the belowground JA cascade. Recent reviews have comprehensively summarized the biosynthesis, signaling, and functional roles of jasmonates in tea plant stress responses [54], further supporting the central role of JA in mediating anti-herbivore defense. This localized activation aligns with established findings in other plant systems, such as *Pinus* species and *Arabidopsis thaliana*, which rapidly deploy JA-mediated defenses upon herbivore challenge [18,19].

The activation of the JA pathway subsequently triggers the downstream phenylpropanoid metabolic cascade, driving the biosynthesis and accumulation of diverse defensive metabolites, including phenolic acids, flavonoids, and phenylamides. This JA-phenylpropanoid synergy is particularly significant in the context of stem-borer defense, as the resulting phenolic compounds not only exhibit direct antimicrobial and insecticidal properties but also contribute to lignification and physical reinforcement of cell walls near the feeding sites [20,21]. The coordinated upregulation of JA biosynthetic genes (*LOX*, *AOS*) and phenylpropanoid pathway genes (*PAL*, *C4H*, *4CL*) observed in this study suggests a tightly coupled signaling-execution module, likely mediated by core transcription factors such as MYC2, which is known to integrate JA signals with downstream metabolic reprogramming [55,56].

The substantial accumulation of phenolic acids (e.g., ferulic acid, p-coumaric acid) in infested roots serves multiple defensive functions. First, these compounds exhibit direct anti-insect activities, including feeding deterrence, growth inhibition, and digestive enzyme interference [20,57]. Second, as precursors of lignin biosynthesis, phenolic acids contribute to cell wall reinforcement, potentially forming physical barriers that restrict larval dispersal within the stem [58]. Third, phenolic acids function as antioxidants that scavenge herbivore-induced reactive oxygen species, thereby mitigating oxidative damage [59,60]. This multi-layered defensive strategy highlights the adaptive significance of activating the phenylpropanoid pathway in response to stem-boring herbivory.

Notably, the significant fluctuations of JA-related metabolites in the roots contrast with the absence of detectable JA accumulation in the leaves, suggesting that *A. induta* infestation elicits a tissue-specific induction of the JA signaling pathway that is restricted to belowground organs. This spatial divergence is likely modulated by the specific feeding site of the herbivore. Because *A. induta* larvae directly damage the woody stems and regions in close proximity to the root system, the roots function as the immediately responding organs and consequently deploy a substantially more intense defense response than the distal foliar tissues [54]. This root-prioritized strategy appears to be an adaptive ecological response, positioning the nearest large storage organ to mount rapid chemical defenses against stem-boring pests.

### 4.3. Foliar and Root Metabolic Coordination Under A. induta Infestation

Although the boring activity of *A. induta* is primarily restricted to woody stems, marked metabolic reprogramming occurred simultaneously in both leaves and roots. This spatial coordination exemplifies a classical systemic defense paradigm in which localized herbivore damage triggers metabolic rewiring in distal, uninfested tissues to coordinate whole-plant immunity [61]. The foliar metabolic response, which is characterized by the preferential accumulation of soluble sugars, sugar acids, and fatty acids, likely reflects a systematic reallocation of photosynthetic products to satisfy the intense energetic and metabolic demands of root defense. Concurrently, these accumulated foliar carbohydrates may operate as vital cross-kingdom signaling molecules that crosstalk with the JA cascade to fine-tune the transcription of downstream defense-related genes [62]. In contrast to the foliar energy reallocation, the roots focus exclusively on launching a direct chemical defense driven by the JA signaling core. This precise spatial partitioning of metabolic labor establishes a highly efficient aboveground-belowground coordinated defense network in tea plants to counteract stem-boring stress [63,64]. A similar multi-omics approach in tea plants against six-spotted spider mite infestation also revealed the coordinated activation of phenylpropanoid and flavonoid biosynthesis pathways, supporting the notion that source–sink metabolite allocation is a common defense strategy in tea plants [65].

Beyond their roles as energetic substrates and long-distance systemic signals, the accumulated foliar carbohydrates likely interact with the belowground JA signaling pathway to mutually optimize or modulate the magnitude of the systemic defense response. Based on our co-expression network topology, the hub genes identified within the root blue and brown modules are hypothesized to sit at critical regulatory nodes that integrate upstream foliar carbohydrate signals with local belowground JA cues. This intersection subsequently licenses the activation of the downstream phenylpropanoid cascade. Future functional validation studies employing stable genetic transformation, such as overexpression or gene silencing techniques, are required to definitively establish the central regulatory roles of these prospective hub genes in mediating aboveground-belowground coordination.

The qRT-PCR validation results further support the transcriptomic alterations observed in the root blue module, showing that the synchronous upregulation of *PAL*, *C4H*, and *4CL* is highly congruent with the RNA-sequencing dataset. Targeted quantitative metabolomic profiles provided orthogonal biochemical evidence confirming the significant accumulation of JA, ferulic acid, and p-coumaric acid. These complementary datasets establish a reliable molecular and biochemical foundation for the proposed defense model, in which the root defense system operates via a JA-signaled core with the phenylpropanoid pathway serving as the primary execution machinery.

### 4.4. Limitations and Future Perspectives

Several caveats in the current study warrant consideration. First, although we characterized distinct shifts in the rhizosphere microbial community, we could not definitively discern whether these alterations represent an active host-mediated defense strategy or merely a passive secondary response to infestation. It is also possible that some of the observed changes in both the microbiome and plant metabolism are influenced by factors other than herbivory, such as tree age-related physiological differences or localized soil heterogeneity. However, the consistency of the infestation-induced responses across five independent biological replicates reduces the likelihood that these patterns are driven solely by stochastic environmental variation. Future research utilizing root exudate back-inoculation experiments will be essential to precisely elucidate the microbial recruitment mechanisms. Second, the multi-omics profiles in this study were captured at a single time point, which precludes a dynamic, time-series resolution of the systemic defense responses. This sampling design was primarily constrained by the unique biological traits of *A. induta*. Specifically, *A. induta* larvae are highly recalcitrant to artificial rearing under laboratory conditions, and their infestation patterns exhibit a strict dependence on host tree age. According to our field surveys, *A. induta* rarely attacks young tea plants, showing an infestation rate of approximately 5% in young orchards. In contrast, the infestation rate reaches 20% in the 30-year-old Shuixian cultivars utilized in this study, and can escalate to 100% in ancient tea trees (unpublished data). This stringent host-age selectivity renders it extraordinarily challenging to conduct multi-time-point dynamic sampling under identical plantation conditions. Future investigations could address this limitation by combining long-term field monitoring of fixed plots or exploring alternative proxy models to capture the temporal dynamics of the defense response. Furthermore, functional validation of the identified hub genes via stable overexpression or gene silencing techniques will represent a crucial next step to conclusively verify their specific roles in tea plant defense. Additionally, while we have interpreted the rhizosphere microbiome shifts as a consequence of the plant‘s defense response, we cannot exclude the alternative explanation that the microbial community changes themselves may contribute to the observed defense phenotypes through microbe–plant signaling interactions. Addressing this possibility will require targeted experiments using synthetic microbial community inoculation and functional metagenomics.

## 5. Conclusions

By integrating rhizosphere microbiomics, metabolomics, and transcriptomics, this study systematically elucidates the distinct tissue-specific metabolic reprogramming patterns in the roots and leaves of tea plants under *A. induta* infestation. Our findings reveal that *A. induta* infestation significantly reshapes the rhizosphere microbial community structure, reducing bacterial diversity and simplifying the co-occurrence network topology. Concurrently, transcriptome and metabolome profiling demonstrated that belowground herbivory vigorously activated the JA signaling pathway in the roots, driving the biosynthesis and accumulation of defensive secondary metabolites, particularly phenolic acids. Conversely, the foliar tissues responded to the belowground challenge via the systematic accumulation of soluble sugars and sugar acids, exhibiting a typical systemic energy reallocation pattern. Integrated transcriptomic–metabolomic analysis further identified the phenylpropanoid biosynthetic pathway as the core regulatory hub of root immunity and uncovered multiple highly correlated gene–metabolite regulatory pairs. Collectively, the integrated soil microbiome–root–leaf coordinated defense network constructed in this study provides novel mechanistic insights into the molecular ecology of tea plant–borer interactions, offering a valuable theoretical foundation for future insect-resistant breeding programs and sustainable integrated pest management strategies.

## Data Availability

The data presented in this study are available on request from the corresponding author.

## Supplementary Materials

The following supporting information can be downloaded at: https://www.mdpi.com/article/10.3390/insects17090911/s1, Table S1: Sequences of primers used for the qRT-PCR assay.

## Abbreviations

The following abbreviations are used in this manuscript:

JA	Jasmonic acid
*C. sinensis*	*Camellia sinensis*
*A. induta*	*Aeolesthes induta*
ISR	induced systemic resistance
SA	salicylic acid
ET	ethylene
*E. grisescens*	*Ectropis grisescens*
PCoA	Principal Coordinate Analysis
H	healthy control
I	infested
